# AstraZeneca COVID‐19 vaccine: A possible risk factor for ischemic stroke and cerebral venous sagittal sinus thrombosis: A case series

**DOI:** 10.1002/ccr3.6017

**Published:** 2022-07-11

**Authors:** Amira Siddig, Khabab Abbasher Hussien Mohamed Ahmed, Mazin S. Hassan Haroun, Abdallah M. Abdallah, Mohamed Malekaldar, Al Hussien Abbasher, Mohammed Abbasher, Abubaker Alsedig Abbasher, Abbasher Hussien

**Affiliations:** ^1^ Faculty of Medicine AlNeelain University Khartoum Sudan; ^2^ Faculty of Medicine University of Khartoum Khartoum Sudan; ^3^ Faculty of Medicine University of Bahri Khartoum Sudan; ^4^ Omdurman teaching Hospital Khartoum Sudan; ^5^ AlYarmouk college Khartoum Sudan; ^6^ Zamzam University College Khartoum Sudan

**Keywords:** AstraZeneca, cerebral venous sinus thrombosis, COVID‐19, CVST, stroke, Sudan, vaccination

## Abstract

One of the most prevalent neurological impairments is cerebrovascular accident (CVA). Ischemic stroke and CVST have been linked to the AstraZeneca COVID‐19 vaccine. Three Sudanese patients developed these diseases after receiving the AstraZeneca COVID‐19 vaccine, indicating a relationship between the AstraZeneca COVID‐19 vaccine and these conditions.

## Abstract 2

Ischemic stroke and CVST have been linked to the AstraZeneca vaccine. It is critical to anticipate such scenarios and educate patients about the potential adverse effects of vaccination in order to encourage them to seek medical treatment early if any symptoms appear after they have been vaccinated to avoid consequences.

## 1 INTRODUCTION

Sudan was the first country in the Middle East and North Africa (MENA) to receive COVID‐19 vaccines from the COVAX facility on March 3, 2021. Approximately 800,000 doses of the AstraZeneca vaccination arrived at Khartoum International Airport.^1^ In this case series, we look at the development of ischemic stroke and cerebral venous sagittal sinus thrombosis in individuals who received the AstraZeneca vaccination without first receiving heparin.

One of the most prevalent neurological impairments is cerebrovascular accident (CVA). Ischemic infarction affects around 85% of patients, while hemorrhagic stroke affects 15%.^2^ The neurological signs vary depending on which region of the Willis circle is damaged (either anterior or posterior circulation problems).^3^ There are controllable and non‐modifiable risk factors for cerebrovascular stroke. Age and sex are non‐modifiable risk factors, whereas diabetes, dyslipidemia, smoking, hypertension, and obesity are all modifiable risk factors.^4^ There is a well‐established link between COVID‐19 and stroke.^5^ The link between thrombotic thrombocytopenia and the COVID‐19 vaccine is still being researched.

The first reports of a link between COVID‐19 vaccinations and thrombotic events came in the media on March 7, 2021, when the Austrian Federal Office for Health Care Safety declared that use of a batch of AstraZeneca ChAdOx1 vaccine had been halted due to thromboembolic occurrences. The UK Medicines and Healthcare Products Regulatory Agency (MHRA) and the Joint Committee on Vaccines and Immunization determined on April 7, 2021, that there was a “possible relationship” between the vaccine and cerebral venous thrombotic events and issued updated recommendations.^6^ By May 18, 2021, two EU/EEA nations had stopped using the vaccine, and 15 had reduced its use to older age groups.^7^ In the United States, cerebral venous thrombosis has been documented after delivery of the AD26.COV2.S Johnson & Johnson (JJ) vaccine, which uses recombinant adenoviral vectors encoding the SARS‐CoV‐2 spike protein like the AstraZeneca ChAdOx1 vaccine.^8^ Thrombosis, thrombocytopenia, and platelet‐activating antibodies to platelet factor 4–polyanion complexes were documented as part of an underlying illness known as “vaccine‐induced immune thrombotic thrombocytopenia” (VITT), or “thrombosis with thrombocytopenia syndrome” (TTS).^9^, ^10^, ^11^ It was realized that thrombocytopenia alone or thrombosis without thrombocytopenia could be part of the spectrum of this condition.

The goal of this case series is to evaluate these symptoms of these patients and look into possible correlations between vaccine administration and the development of thrombosis and thrombocytopenia.

## 2 CASES PRESENTATION

### 2.1 Case 1

History: A 45‐year‐old Sudanese man was brought to our neurology private clinic seven (in August 2021) days after receiving the COVID‐19 AstraZeneca Vaccine with a sudden episode of left‐sided paralysis. Headache and neck pain had preceded the disease.

The patient's neurological history was taken in detail. Hypertension, diabetes, heart disease, renal illness, and hepatic disease were all ruled out based on the patient's medical history.

Examination: All systems were examined and found to be normal. He was perplexed. The blood pressure reading was 180/75. Neck stiffness and extensive bilateral papilledema were the only anomalies in his central nervous system. Power grade zero, hypotonia, and areflexia were found in both the upper and lower left limbs.

Investigations: Investigations revealed he had significant thrombocytopenia, low platelets (30 × 10^3^), a high D‐dimer, and a total blockage of the right carotid artery on a Carotid Doppler scan. Sagittal sinus thrombosis and right cerebral infarction were discovered on brain MRI and MRV.

**FIGURE 1:**
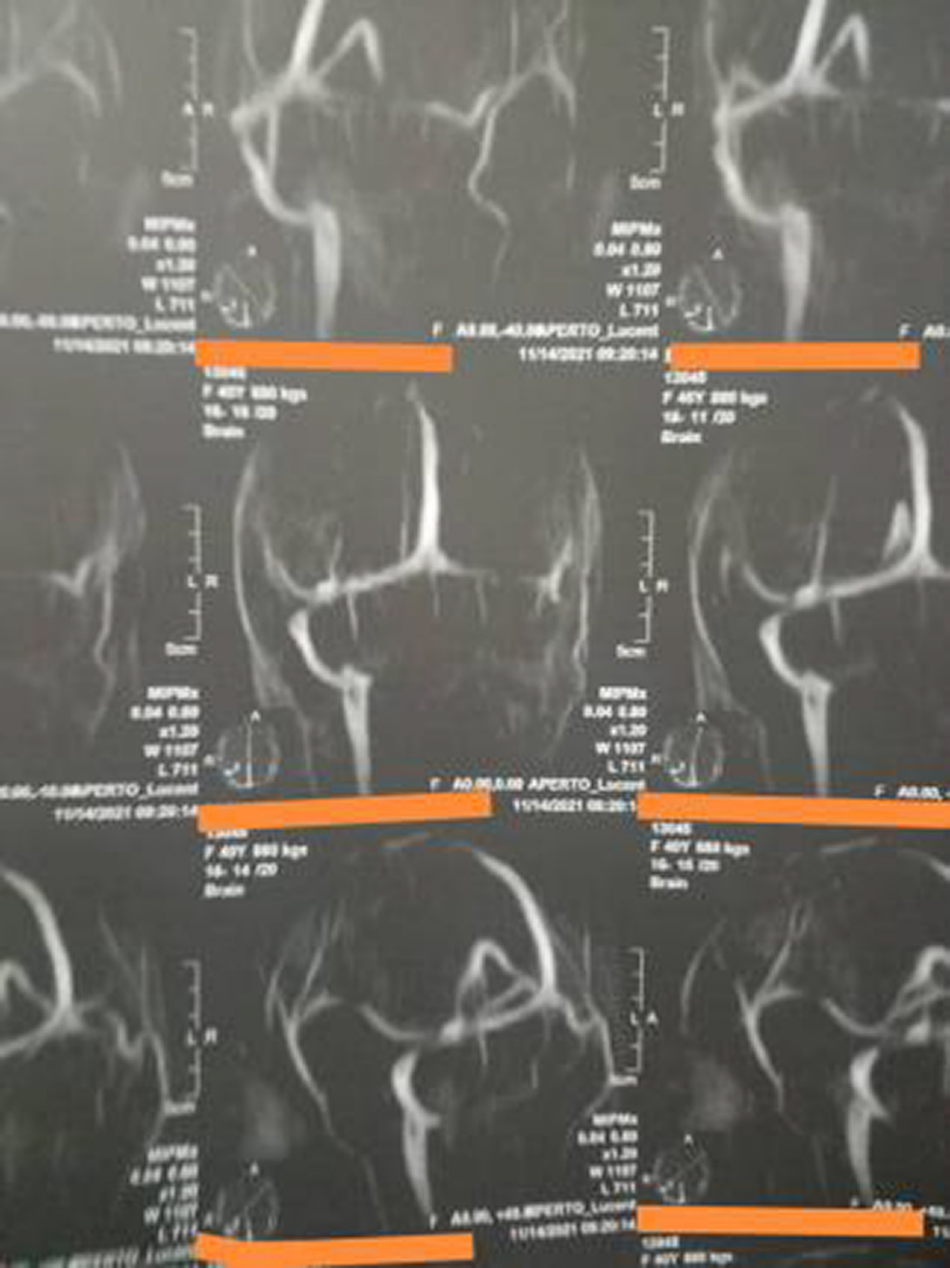
MRV revealing sagittal sinus thrombosis

Treatment: In August 2021, he was admitted to the National Center for Neurological Science, a tertiary neurology hospital. Regarding this case, we consulted a senior neurosurgeon—because of the concerns raised due to the sagittal sinus thrombosis—and he did not recommend doing an emergent decompressive craniotomy, as it is recommended for patients with CVST who are experiencing impending herniation.^12^ Despite receiving intravenous immunoglobulin, methylprednisolone, and platelet transfusions during his stay, he experienced convulsions. The patient passed away 2 days after being admitted. (Figure 1).

### 2.2 Case 2

History: After 10 days of receiving the COVID‐19 AstraZeneca vaccination, a 57‐year‐old Sudanese woman was brought to our private neurology clinic with left‐sided weakness in September 2021. There were no headaches, convulsions, or loss of consciousness prior to the condition. Hypertension, diabetes, heart disease, renal illness, and hepatic disease were all ruled out based on the patient's medical history.

Examinations: She was sick on inspection, but not pale, jaundiced, or cyanosed. The heart rate was 80 beats per minute, and the blood pressure was 110/70 millimeters of mercury. A study of the central nervous system revealed signs of left‐sided hemiparesis (power was grade three with hypotonia and areflexia in both upper and lower limbs). All of the sense modalities were intact.

Investigations: A CT brain scan revealed a right cerebral infarction, whereas a Carotid Doppler scan revealed a partial obstruction of the left carotid artery. Platelets were extremely low (35 × 10^3^), yet D‐dimer levels were quite high.

**FIGURE 2:**
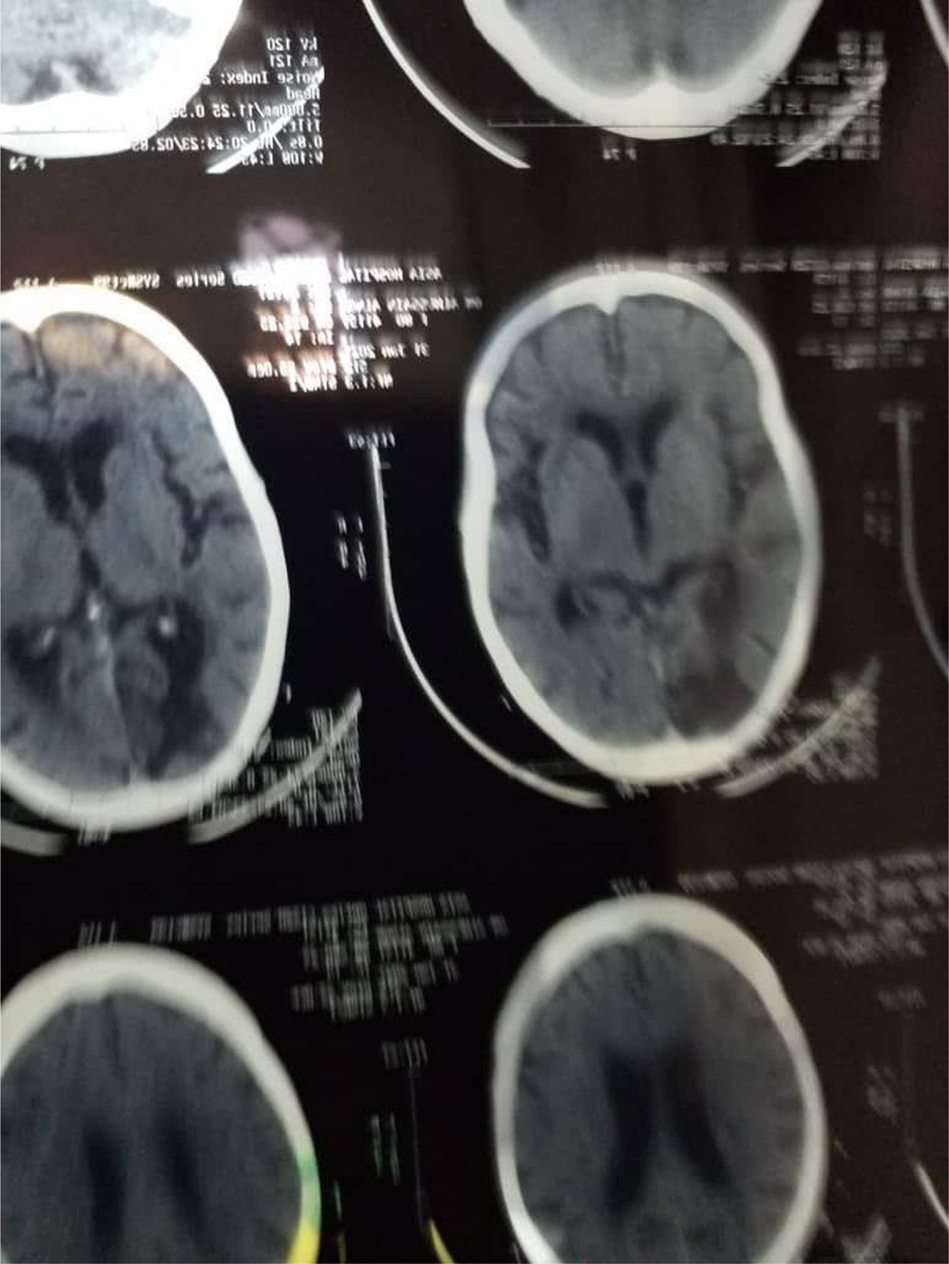
CT brain showed cerebral infarction

Treatment: In September 2021, she was admitted to the National Center for Neurological Science (a tertiary neurology hospital). She underwent methylprednisolone, intravenous immunoglobulin, and platelet transfusions while in the hospital. She made significant progress and was discharged home with a power grade of four and the ability to walk without assistance after 2 weeks. (Figure 2).

### 2.3 Case 3

History: A 65‐year‐old Sudanese man visited our private neurology clinic after suffering from numerous transient ischemic episodes (TIAs). He had got the COVID‐19 AstraZeneca Vaccine thirteen days prior to the filing of his complaint. Hypertension, diabetes, heart disease, renal illness, and hepatic disease were all ruled out based on the patient's medical history.

Examinations: There were no abnormalities found throughout a thorough system assessment.

Investigations: D‐dimer was high, platelets were low (65 × 10^3^), and the Carotid Doppler scan was normal, according to the inquiry. Periventricular ischemia was discovered on MRI (small vessel disease).

Treatment: In October 2021, he was admitted to the National Center for Neurological Science, a tertiary neurology hospital. During his stay in the hospital, the patient was given methylprednisolone and platelets. After 1 week on aspirin and atorvastatin, he exhibited significant improvement and was discharged.

## 3 DISCUSSION

COVID‐19 is associated with common neurological diseases including stroke. Stroke is the most common neurological disorder. It is the third killer worldwide and is one of the commonest causes of disability. There are two main types of stroke: Ischemic stroke (constitutes 85%) and hemorrhagic stroke (constitutes 15%).^13^

COVID‐19 infection is an acute inflammatory condition associated with increased incidence of fatty plaques formation and injury of endothelial cells of the vascular wall. Coagulopathy and vascular endothelial dysfunction have been proposed as complications of COVID‐19.^14^ The coexistence of inflammation, hypoxia, and hypercoagulability can lead to formation of microthrombi and macrothrombi in vessels. So, patients with COVID‐19 are at an increased risk of venous and arterial thromboembolization leading to cerebrovascular accidents.^15^

COVID‐19 viruses have caused increased morbidity and mortality worldwide. To meet this extraordinary challenge, new vaccines have been developed with a speed that never seen before in medical history; vaccination against SARS‐COV‐2 is considered an effective preventive strategy to halt the COVID‐19 pandemic. Several vaccines against COVID‐19 have been developed.^16^

Recently, reports of coagulopathy have appeared associated with COVID‐19 vaccinations and particularly the ChAdOx1 nCoV‐19 vaccine.

Immune thrombotic thrombocytopenia is a rare side effect of vaccination against COVID‐19 referred to as vaccine‐induced immune thrombotic thrombocytopenia (VITT). VITT usually occurs 1–2 weeks after vaccination with ChAdOx1 nCoV‐19. Cerebral venous thrombosis is more common than arterial thrombosis.^17^

The following definition for VITT was proposed: patients presenting with acute thrombosis and thrombocytopenia with elevated D‐dimers, using a D‐dimer threshold of <2000 μg/L for VITT‐unlikely and > 4000 μg/L for VITT‐suspected. They showed that 22 (96%) of 23 patients with VITT had antibodies against platelet factor 4 (PF4)^18^

After the introduction of the adenovirus vector vaccine ChAdOx1 (Oxford–AstraZeneca), we report three cases of thrombosis with thrombocytopenia, each started 8–14 days after administration of the first vaccine dose.

A mechanism similar to heparin‐induced thrombocytopenia was proposed with antibodies to platelet factor 4 (PF4). The thrombosis is likely caused by platelet‐activating antibodies against PF4 produced after vaccination. It has been termed as vaccine‐induced prothrombotic immune thrombocytopenia to differentiate it from heparin‐induced thrombocytopenia.^19^

Thrombosis and thrombocytopenia were subsequent events for our vaccinated patients without any administration of heparin. Since this disease resembles autoimmune heparin‐induced thrombocytopenia clinically, we were supposed to refer the serum of the patients for an investigation of platelet‐activating antibodies against platelet factor 4 (PF4)‐heparin immediately, but were unable to do so due to lack of availability. Although it is unnecessary to wait for laboratory diagnosis before making treatment decisions—such as administering intravenous immune globulin or commence anticoagulation—yet, detection of these unusual platelet‐activating antibodies will be important for identifying cases and determining the risk–benefit ratio of this vaccine and others in the future.^10^ Also, the fact that adenovirus attaches to platelets and activates them is widely established.^20^ In a similar study, the quantity of adenovirus in a 500‐microliter vaccination injection given 1 or 2 weeks prior did not have a role in the later platelet activation seen in patients. Interactions between the vaccination and platelets, or the vaccine and PF4, on the contrary, might have a role in pathogenesis. Free DNA in the vaccination might be a cause for these PF4‐reactive antibodies.^10^ Yet, it is important to note that the existence of an association may not necessarily imply cause and effect, especially when the incidence of thromboembolic events observed in vaccinated patients is lower than expected compared with the incidence of events in the general population.^21^

Regarding treatment of the three cases, our treatment plan included different combinations (which are the new regimen for treating thrombocytopenia) according to the status of the patient—of methylprednisolone, intravenous immunoglobulin, platelet transfusion, aspirin, and atorvastatin—which is similar to a case series where a similar treatment plan was established successfully.^22^

Literature describes how Neurosurgical Considerations regarding decompressive craniotomy can be used to treat cerebral hemorrhage after SARS‐CoV‐2‐vaccination in VITT.^23^ One possibility for a surgical treatment is a unilateral hemi‐craniotomy, which would concentrate on the large hematoma and/or infarction.^24^

Regarding our first case, we consulted a senior neurosurgeon—because of the concerns raised due to the sagittal sinus thrombosis—and he did not recommend doing an emergent decompressive craniotomy, as it is recommended for patients with CVST who are experiencing impending herniation.^12^ Given the laboratory results of the patient, the clinical picture (CVST as a complication of VTIT) and the lack of available modalities (for further investigations), we assumed the patient to have VITT which is similar to HIT, and we treated him accordingly. So our treatment plan was focused on increasing platelet count in order to stabilize the patient, giving intravenous immunoglobulin, methylprednisolone, and platelet transfusions during his stay. Unfortunately, the patient still passed away. We scheduled a follow‐up plan for the other cases, as drug dosage regimens—which is a common application of therapeutic drug monitoring—in similar cases is recommended as a part of follow‐up.^25^

In order to prevent infection and complications resulting from this virus, vaccination seems to be the most effective method. In light of our findings, it is important for physicians to request ELISA tests for PF4/polyanion, including functional confirmation tests, in patients who experience unexpected symptoms after vaccination. Patients with higher risks of thrombosis should be evaluated before vaccination and followed up after vaccination. If the suspension level is not high, we do not have to alert patients. This study was done on a very small sample; therefore, it is not possible to generalize the inferences drawn.

To our knowledge, this is the first case series done in Sudan regarding this topic. It is Important to consider such cases and educate patients about possible side effects of vaccination, in order to encourage them to seek medical help if any symptoms start to develop after getting vaccinated as early as possible to reduce chances of severe disease, and to give prophylactic heparin in high‐risk cases.

## 4 CONCLUSION

According to what was proposed in this study, ischemic stroke and cerebral venous thrombosis and especially sagittal sinus thrombosis are the most frequent types of stroke that tend to appear after vaccination. Vaccine‐related arterial thrombosis in the brain is exceedingly rare. Ischemic stroke was associated with large artery occlusion, both carotid and middle cerebral artery. The thrombosis is likely caused in a way similar to heparin‐induced thrombocytopenia; platelet‐activating antibodies against PF4 produced after vaccination; hence, laboratory tests are needed for definitive diagnosis.

## AUTHOR CONTRIBUTIONS

All authors participated in planning the study, data collection, results, and discussion sections. AS, KA, MSH, and AM collected data and wrote first draft. MM, AAH, MA, AH, and AA reviewed and wrote the final draft.

## CONFLICT OF INTEREST

The authors have no conflict of interest to declare.

## ETHICAL APPROVAL

Ethical approval was obtained from each center ethical committee. Both verbal and written consents to publish this information were obtained from the patients.

## CONSENT

Written informed consent was obtained from the patient to publish this report in accordance with the journal's patient consent policy. All authors gave their consents to publish this manuscript.

## Data Availability

The data that support the findings of this study are available from the corresponding author upon reasonable request.

## References

[1] Sudan Receives the First Delivery of COVID‐19 Vaccines with over 800,000 Doses. UNICEF; 2021. https://www.unicef.org/press‐releases/sudan‐receives‐first‐delivery‐covid‐19‐vaccines‐over‐800000‐doses

[2] Tadi P , Lui F . Acute Stroke. [Updated 2021 Sep 29]. In: StatPearls [Internet]. Treasure Island (FL): StatPearls Publishing; 2021. Available from: https://www.ncbi.nlm.nih.gov/books/NBK535369/?report=classic

[3] Kuriakose D , Xiao Z . Pathophysiology and treatment of stroke: present status and future perspectives. Int J Mol Sci. 2020;21(20):7609. 10.3390/ijms21207609 PMC7589849 33076218

[4] Boehme A , Esenwa C , Elkind M . Stroke risk factors, genetics, and prevention. Circ Res. 2017;120(3):472‐495. 28154098 10.1161/CIRCRESAHA.116.308398 PMC5321635

[5] Stein L , Mayman N , Dhamoon M , Fifi J . The emerging association between COVID‐19 and acute stroke. Trends Neurosci. 2021;44(7):527‐537. 33879319 10.1016/j.tins.2021.03.005 PMC8026270

[6] Medicines and Healthcare products Regulatory Agency. MHRA issues new advice, concluding a possible link between COVID‐19 Vaccine AstraZeneca and extremely rare, unlikely to occur blood clots. GOVUK; 2021. Available: https://www.gov.uk/government/news/mhra‐issues‐new‐advice‐concluding‐a‐possible‐link‐between‐covid‐19‐vaccine‐astrazeneca‐and‐extremely‐rare‐unlikely‐to‐occur‐blood‐clots

[7] ECDC . Overview of EU/EEA country recommendations on COVID‐19 vaccination with Vaxzevria, and a scoping review of evidence to guide decision‐making. 2021. Available: https://www.ecdc.europa.eu/sites/default/files/documents/Overview%20EU%20EEA%20country%20recommendations%20on%20COVID‐19%20vaccination%20Vaxzevria%20and%20scoping%20review%20of%20evidence.pdf

[8] See I , Su JR , Lale A , et al. US case reports of cerebral venous sinus thrombosis with thrombocytopenia after Ad.26COV2.S vaccination, March 2 to April 21, 2021. JAMA. 2021;325:2448‐2456. doi:10.1001/jama.2021.7517 33929487 PMC8087975

[9] Schultz NH , Sørvoll IH , Michelsen AE , et al. Thrombosis and Thrombocytopenia after ChAdOx1 nCoV‐19 Vaccination. N Engl J Med. 2021;384:2124‐2130. doi:10.1056/NEJMoa2104882 33835768 PMC8112568

[10] Greinacher A , Thiele T , Warkentin TE , Weisser K , Kyrle PA , Eichinger S . Thrombotic thrombocytopenia after ChAdOx1 nCov‐19 vaccination. N Engl J Med. 2021;384:2092‐2101. doi:10.1056/NEJMoa2104840 33835769 PMC8095372

[11] Chen RT , Black S . Interim Case Definition of Thrombosis with Thrombocytopenia Syndrome (TTS). Brighton Collaboration; 2021. https://brightoncollaboration.us/thrombosis‐with‐thrombocytopenia‐syndrome‐interim‐case‐definition/

[12] Coutinho JM , Majoie CB , Coert BA , Stam J . Decompressive hemicraniectomy in cerebral sinus thrombosis: consecutive case series and review of the literature. Stroke. 2009;40(6):2233‐2235. doi:10.1161/STROKEAHA.108.543421 19372443

[13] Sidig A , Salah‐Eldien M , Mohamed Ahmed K , et al. COVID‐19 as a risk factor for ischemic stroke, a case report, khartoum, sudan, 2020. Acta Scientific Neurology. 2021;4(11):18‐21.

[14] Del Turco S , Vianello A , Ragusa R , Caselli C , Basta G . COVID‐19 and cardiovascular consequences: Is the endothelial dysfunction the hardest challenge? Thromb Res. 2020;196:143‐151. 32871306 10.1016/j.thromres.2020.08.039 PMC7451195

[15] Wijeratne T , Gillard Crewther S , Sales C , Karimi L . COVID‐19 pathophysiology predicts that ischemic stroke occurrence is an expectation, not an exception—a systematic review. Front Neurol. 2021;11:607221. 33584506 10.3389/fneur.2020.607221 PMC7876298

[16] Kyriakidis N , López‐Cortés A , González E , Grimaldos A , Prado E . SARS‐CoV‐2 vaccines strategies: a comprehensive review of phase 3 candidates. npj Vaccines. 2021;6(1):28. 33619260 10.1038/s41541-021-00292-w PMC7900244

[17] Kelton J , Arnold D , Nazy I . Lessons from vaccine‐induced immune thrombotic thrombocytopenia. Nat Rev Immunol. 2021;21(12):753‐755. 34663971 10.1038/s41577-021-00642-8 PMC8521503

[18] Perry R , Tamborska A , Singh B , et al. Cerebral venous thrombosis after vaccination against COVID‐19 in the UK: a multicentre cohort study. The Lancet. 2021;398(10306):1147‐1156. 10.1016/S0140-6736(21)01608-1 PMC8346241 34370972

[19] Huynh A , Kelton J , Arnold D , Daka M , Nazy I . Antibody epitopes in vaccine‐induced immune thrombotic thrombocytopaenia. Nature. 2021;596(7873):565‐569. 34233346 10.1038/s41586-021-03744-4

[20] Stone D , Liu Y , Shayakhmetov D , Li ZY , Ni S , Lieber A . Adenovirus‐platelet interaction in blood causes virus sequestration to the reticuloendothelial system of the liver. J Virol. 2007;81(9):4866‐4871. doi:10.1128/JVI.02819-06 17301138 PMC1900148

[21] European Medicines Agency Pharmacovigilance Risk Assessment Committee . Signal assessment report on embolic and thrombotic events (SMQ) with COVID‐19 Vaccine (ChAdOx1‐S [recombinant])–Vaxzevria (previously COVID‐19 Vaccine AstraZeneca)(Other viral vaccines).

[22] Al‐Mayhani T , Saber S , Stubbs MJ , et al. Ischaemic stroke as a presenting feature of ChAdOx1 nCoV‐19 vaccine‐induced immune thrombotic thrombocytopenia. J Neurol Neurosurg Psychiatry. 2021;92(11):1247‐1248. doi:10.1136/jnnp-2021-326984 34035134

[23] Gessler F , Schmitz AK , Dubinski D , et al. Neurosurgical Considerations Regarding Decompressive Craniectomy for Intracerebral Hemorrhage after SARS‐CoV‐2‐Vaccination in Vaccine Induced Thrombotic Thrombocytopenia‐VITT. J Clin Med. 2021;10(13):2777. Published 2021 Jun 24. 10.3390/jcm10132777 34202817 PMC8269113

[24] Güresir E , Vatter H , Schuss P , et al. Rapid closure technique in decompressive craniectomy. J Neurosurg. 2011;114(4):954‐960. doi:10.3171/2009.12.JNS091065 20113157

[25] Platt DR . Individualization of drug dosage regimens. Clin Lab Med. 1987;7(2):289‐299. 3301171

